# Encephalopathy in a Young Female With COVID-19: A Case Report

**DOI:** 10.7759/cureus.37373

**Published:** 2023-04-10

**Authors:** Andrew Kim, Cristian Valdez, Weston Truman, George Trad, Cordelia Solomon, Yi McWhorter

**Affiliations:** 1 Internal Medicine, MountainView Hospital, Las Vegas, USA; 2 Intensive Care Unit, MountainView Hospital, Las Vegas, USA

**Keywords:** corticosteroids, autoimmune encephalopathy, autoimmune encephalitis, acute encephalitis, covid-19

## Abstract

Cases of severe central nervous system (CNS) complications have been reported in relation to coronavirus-19 (COVID-19). Cases of encephalitis have been reported primarily in older patients with multiple comorbidities. We present a case of encephalitis in a young female patient with a history of chronic marijuana use that presented with nausea, vomiting, and acute altered mental status. Extensive testing for infectious and autoimmune causes of encephalitis were negative, except for a positive COVID-19 test. She was treated with steroids and intravenous immune globulin (IVIG) and improved with residual mutism.

## Introduction

In the winter of 2019, reports of a novel coronavirus strain causing severe pneumonia began spreading across the world. Coronavirus-19 (COVID-19) was quickly associated with neurologic symptoms such as dizziness, headache, anosmia, and dysgeusia [1]. Shortly after the World Health Organization (WHO) declared a pandemic on March 11, 2020, reports of encephalitis and seizure associated with COVID-19 infection began to arise [2].

The first reports of encephalitis were seen in a patient in China, shortly followed by reports of a 24-year-old male in Japan presenting with necrotizing encephalopathy from COVID-19 infection [2]. Encephalitis is now known to have a strong association with COVID-19 infection and is known to be a predictive factor in increased length of stay and mortality [3]. Here, we present a young female with acute encephalopathy due to COVID-19 infection that resulted in lingering psychiatric dysfunction.

## Case presentation

A 23-year-old female with a history of chronic marijuana use was brought into the emergency room after an episode of nausea and vomiting followed by lethargy and altered mental status, as reported by her significant other. She had a similar episode a few months prior but did not have altered mental status at that time. Blood count and metabolic panel were normal. Thyroid-stimulating hormone (TSH) was normal as well. Immunology labs were notable for an elevated immunoglobulin G (IgG) level at 2144 mg/dl and a slight decrease of IgA at 82 mg/dl with normal immunoglobulin M (IgM) level. The original diagnosis for this patient was seizure caused by marijuana use laced with possible unknown substances, as her last marijuana use was the night before her emergency room visit. However, urinalysis was normal, and the drug screen was negative. Computed tomography (CT) without contrast of the brain showed no acute processes. Magnetic resonance imaging (MRI) of the brain showed nonspecific abnormal findings with a wide differential favoring infection or metabolic encephalopathy. The patient was found to be positive for COVID-19 infection upon admission.

The patient was upgraded to the intensive care unit (ICU) due to increased confusion and worsening respiratory status that required mechanical ventilation support. While in the ICU, an electroencephalogram (EEG) showed two episodes of heavy seizure burden. The patient was intubated to protect her airway after the episodes of seizure and she was started on levetiracetam. A lumbar puncture was done (Table 1) and showed no acute bacterial, viral, or fungal infections of the cerebrospinal fluid. She was started on a high-dose steroid course as well as intravenous immune globulin (IVIG).

**Table 1:** Cerebrospinal fluid (CSF) fluid results Abnormal results are indicated with a (*). Normal white blood cell count suggests no infectious process.

Type	Result	Units	Reference
Volume	15	ml	
Appearance	Clear		Clear
Color	Colorless		Colorless
WBC	0.004	x10^3^ul	0.005
RBC	<0.002	x10^6^ul	0.000
Glucose	75*	mg/dl	45-70
Total protein	137*	mg/dl	15-45
Myelin basic protein	7.4*	ng/ml	0.0-2.9

Titers for *Bartonella*, Lyme disease, *Brucella*, *Coccidioides*, West Nile virus, Hepatitis, human immunodeficiency virus, and tuberculosis were negative. Additionally, antibodies for anti-neutrophil, Sjogren's, Smith, Purkinje Cell (PCA-2), and amphiphysin were negative as well. The patient was ultimately diagnosed with autoimmune encephalitis due to COVID-19 infection after excluding other possible causes of her condition.

The patient improved after her course of steroids and IVIG and was extubated. EEG was negative for seizure after initiation of levetiracetam and she did not have any more seizures after the initial two episodes. The patient's mental status was not at baseline upon extubation but slowly improved throughout the hospital stay. The patient was nonverbal throughout the remainder of her hospital stay but responded to questions appropriately with head nods and shakes. She was discharged to an acute rehabilitation facility with plans to follow up with neurology and psychiatry outpatient.

## Discussion

Encephalitis is a known complication of COVID-19, but it is rarely seen in young patients with no comorbidities. In a systematic review, the incidence rate of encephalitis in COVID-19 patients was 0.215%, with an average onset of 14.5 days between diagnosis of COVID-19 and encephalitis onset [4]. Another systematic review showed that encephalitis was more common in males, and the average age was 49.3 years old, with a mortality rate of 13.4-20.0% [4,5]. Seizures were seen in 29.5% of patients who presented with COVID-19-related encephalitis [5]. The presentation of our patient was indeed abnormal, as she presented with acute encephalitis at the age of 23. However, we cannot be certain of the exact date of onset of her COVID-19 infection. Although her family denied symptoms prior to her acute confusion, she could have been infected and asymptomatic.

The exact mechanism of COVID-19-related encephalitis is unknown. One possible mechanism is via infection of endothelial cells via the angiotensin-converting enzyme 2 receptor found on vascular endothelium [6]. Another possibility is by causing an inflammatory state that affects the blood-brain barrier’s permeability, leading to an immune-mediated inflammation of the central nervous system (CNS) [7]. Because our patient had an elevated IgG level, her case was most likely due to the latter theory.

Treatment for COVID-19 encephalitis is mostly supportive. Our patient received a course of steroids and IVIG that seemed to improve her condition, making it more likely that her encephalitis was immune-mediated.

## Conclusions

Encephalitis in hospitalized patients can present with a variety of symptoms, making accurate diagnosis difficult. This case serves to highlight the importance of maintaining a broad differential in the setting of altered mental status. It emphasizes the need to combine clinical presentation with lab findings in order to appropriately diagnose the cause of encephalitis and lends further emphasis on the many complications we continue to find associated with the COVID-19 pandemic. 
